# How much sweating is enough? Predicting thermoregulatory capacity in patients with ectodermal dysplasia based on a minimal pilocarpine-induced sweat volume

**DOI:** 10.3389/fped.2026.1934287

**Published:** 2026-08-18

**Authors:** Anja Bachfischer, Carola Berking, Smail Hadj-Rabia, Umberto Simeoni, Anna L. David, Holm Schneider

**Affiliations:** 1 Center for Ectodermal Dysplasias, University Hospital Erlangen, Erlangen, Germany; 2 Department of Pediatrics, Friedrich-Alexander-Universität Erlangen-Nürnberg, Erlangen, Germany; 3 Department of Dermatology, Friedrich-Alexander-Universität Erlangen-Nürnberg, Erlangen, Germany; 4 Department of Dermatology, Reference Centre for Genodermatoses and Rare Skin Diseases, Hopital Necker-Enfants malades, University of Paris-Cité, Paris, France; 5 Division of Pediatrics, University of Lausanne, Lausanne, Switzerland; 6 EGA Institute for Women’s Health, University College London, London, United Kingdom

**Keywords:** clinical trial endpoint, hypohidrotic ectodermal dysplasia, hypomorphic mutation, Sweat volume, thermoregulation

## Abstract

**Background:**

Pathogenic *EDA* gene variants underlying X-linked hypohidrotic ectodermal dysplasia cause either anhidrosis (null mutations) or hypohidrosis (hypomorphic mutations). Due to their inability to sweat, heat-stressed individuals with *EDA* null mutations are unable to regulate their body temperature without external cooling and are thus vulnerable to heat-induced injury. For the purpose of developing a therapeutic intervention it would be necessary to know what sweat production is sufficient for normal thermoregulation, e.g., by correlating perspiration in a standard test with thermoregulatory ability, so that a certain sweat volume can be used as an endpoint in clinical trials of drug candidates.

**Methods:**

In patients with hypomorphic *EDA* mutations, perspiration in response to pilocarpine iontophoresis was determined with the Macroduct sweat collector and volumetry after 30 minutes. Parental- or self-reported heat intolerance and clinical signs of overheating were recorded. If sweat production was borderline, exercise-induced and thermal sweating were assessed by infrared thermography of the skin and core temperature monitoring. In addition, a systematic review of the literature reporting sweat volumes obtained after pilocarpine iontophoresis and thermoregulatory ability of patients with hypomorphic *EDA* mutations was performed.

**Results:**

We studied a rare cohort of individuals with hypomorphic *EDA* mutations (*n* = 14) managed at the German national centre for ectodermal dysplasias. Subjects who produced more than 15 µL of sweat upon stimulation with pilocarpine did not show heat intolerance or signs of overheating. Pilocarpine-induced sweating of 16 µL/30 min in early childhood was found to correlate with normal thermoregulation at the age of 10 years. The literature review suggested that a pilocarpine-induced sweat production of 15 µL/30 min in boys indeed represents the bottom of the normal range.

**Conclusion:**

A sweat volume ≥15 µL, measured in a standard test on male infants using the Macroduct system, appears to indicate normal thermoregulatory capacity in the European climate.

## 1 Introduction

Adequate perspiration is essential for thermoregulation, especially in hot environments and during intense physical activities or febrile illnesses. The lack or complete absence of sweat glands as observed in patients with X-linked hypohidrotic ectodermal dysplasia (XLHED; MIM 305100) leads to lifelong medical problems, including dangerous episodes of overheating (1–4). XLHED is a rare congenital genetic disorder caused by the deficiency of ectodysplasin A1, a signaling protein encoded by the gene *EDA* (5), during intrauterine development. It was estimated to affect approximately 4 per 100,000 live male births (6).

A standard topical pilocarpine iontophoresis with subsequent sweat collection over 30 minutes using the Macroduct system can be used to confirm hypo- or anhidrosis, a cardinal feature of XLHED, thereby enabling a clinical diagnosis to be established (7). As assessed in this standard test, Schneider et al. (8) recently reported sweat volumes in healthy individuals screened for cystic fibrosis. In the youngest group (< 6 months of age); minimum sweat volumes of girls and boys produced were 11 and 15 µL, respectively; the normal range was quite large (up to 82 µL) with means of 39 µL in boys and 41 µL in girls (8). This may be explained by variable fluid intake, differences in room temperature, use of creams or lotions on the skin prior to the test, and genetic factors that are not known in healthy individuals, such as filaggrin gene variants which may influence sweating. Similar ranges were found by Hadj-Rabia et al. (9) in healthy infants of the same age (95 boys and 90 girls). In older children, pilocarpine-induced sweat volumes did not differ significantly until the onset of puberty, when perspiration increased in male subjects (8, 9). Given that body surface area expands principally during childhood and that the total number of eccrine sweat glands remains constant throughout, it can be concluded that the older a person is, the more effectively the sweat glands work. In addition, individual characteristics, local body functions and ethnicity are known to underlie differences in sweating (10–12).

Pathogenic *EDA* variants cause either anhidrosis (null mutations) or hypohidrosis (hypomorphic mutations) (7). Most individuals with XLHED carry *EDA* null mutations and have been completely unable to perspire since birth. XLHED can be detected prenatally, where noninvasive ultrasound examination of fetal tooth bud development is both reliable and convenient (13, 14). This technique is emerging as a recognised diagnostic procedure (15). In infants with *EDA* null mutations, the prenatal administration of a replacement protein for the missing ectodysplasin-A molecule was shown to result in babies born with normal sweating ability (16). A clinical trial is currently being conducted to investigate the efficacy and safety of intra-amniotic administration of the replacement protein to fetal male subjects with XLHED (17). Its primary endpoint is the mean sweat volume obtained within 30 minutes after pilocarpine iontophoresis, using the Macroduct collection system on both forearms at 6 months of age. The results will be directly compared with the similarly collected sweat volumes from genotype-matched controls: either untreated affected male relatives or matched controls from an external XLHED database. Key to the determination of clinical efficacy in the ongoing trial is the correlation of normal thermoregulation with the sweat volumes collected under the prescribed standard conditions. In short: “What sweat production is sufficient for normal thermoregulation?”. Of particular importance would be the minimal pilocarpine-induced sweat volume associated with the absence of hyperthermic episodes under normal circumstances. To answer this question we analysed data from a prospective cohort study involving patients with hypomorphic *EDA* mutations. We also performed a systematic review of the scientific literature about sweat production and thermoregulation in patients with such *EDA* variants. Our aim was to explore the correlation between measured sweat volumes and the ability to thermoregulate normally in individuals whose sweat volumes are at the bottom of the established normal range.

## 2 Methods

### 2.1 Cohort study

We investigated a cohort of individuals with hypomorphic *EDA* mutations who participated in the EdeReaLife study (started in July 2023). This is an observational, multicentre, prospective study of the natural history and disease burden of XLHED. Patients are followed over two years from inclusion. All available data on sweat production and thermoregulation prior to enrolment were collected. No specific treatment was required to be eligible for inclusion, nor was it recommended within the study period as participants continued to be treated according to local best practice. In connection with the EdeReaLife study, the records of all patients with hypomorphic *EDA* mutations known at the Centre for Ectodermal Dysplasias in Erlangen (ethnically Central or Western European) were consulted and the results therein were compiled and analysed.

**Figure 1:**
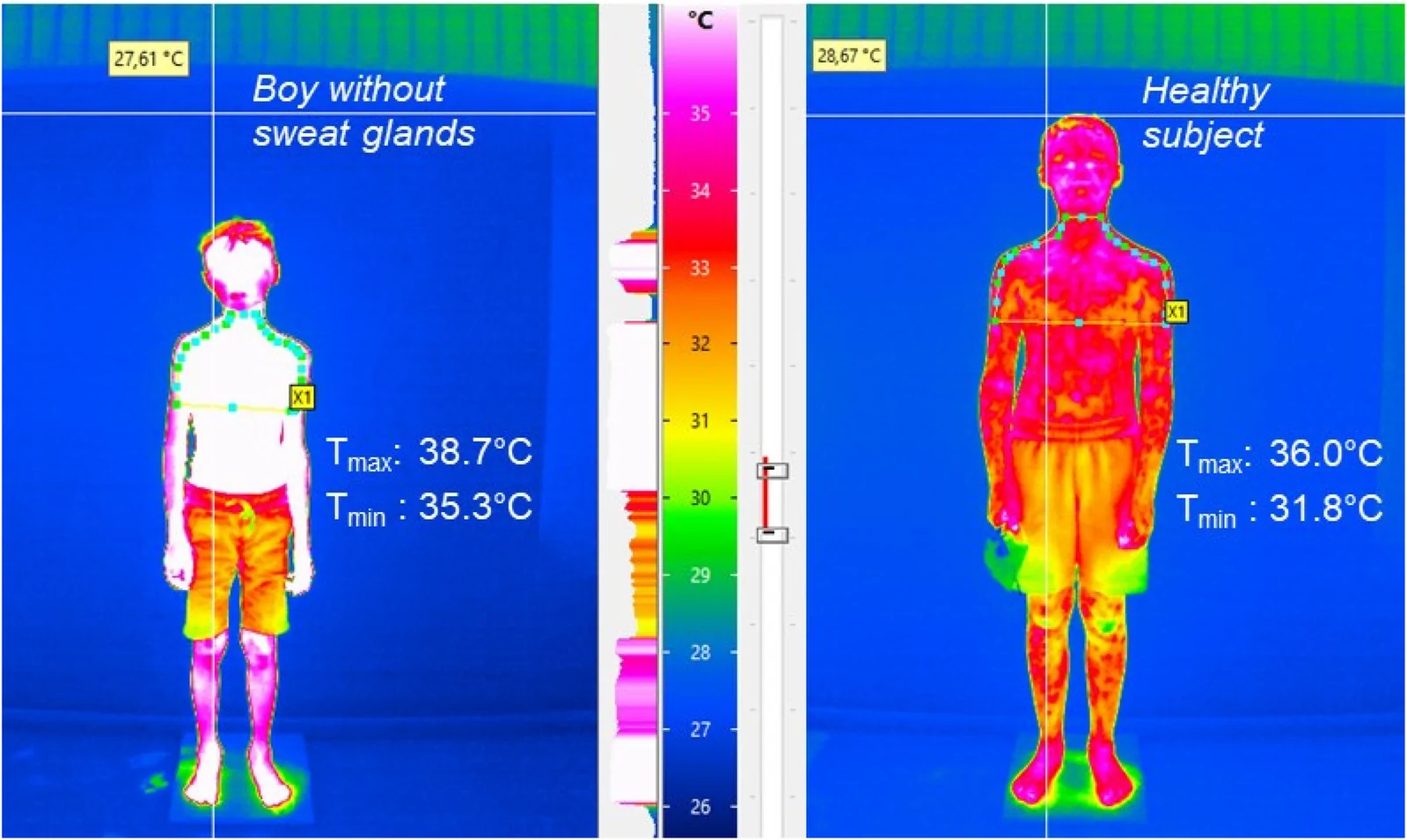
Infrared thermography of a boy without sweat glands and a healthy child after exercising for 20 minutes. A boy with XLHED and a healthy boy of the same age were both examined to obtain medical clearance for sports activities. In the healthy child, efficient sweating prevented the exercise-induced heat built-up that was observed in the boy with XLHED. Note the temperature difference of almost 3°C on the skin.

Assessment of pilocarpine-induced sweat production and visualization of exercise-induced or thermal sweating by infrared thermography (Figure 1) were performed as described previously (8).

Body core temperature measurements were made with an ingestible thermometer pill (CorTemp; HQ Inc., Palmetto, USA). External recording of body core temperature was started approximately 30 minutes after the ingestion of a calibrated pill which transmitted temperature data continuously for several hours. Basal values were noted when temperature fluctuations had ceased indicating that body temperature had reached a steady value. The subject was then told to start exercising on a bicycle ergometer with continuous heart rate monitoring and increasing physical strain (0.5 W/kg body weight at the beginning, raised every 4 minutes by 0.5 W/kg body weight). Criteria for discontinuation were a body core temperature of more than 40°C, an increase in heart rate above an age-adjusted threshold, or subjective exhaustion. Body core temperature was recorded immediately before the start of exercising and then at 30, 40, 60 and 90 minutes after the initiation of exercising.

### 2.2 Systematic literature review

A literature search of databases including PubMed, Google Scholar, and Embase (for published articles) and bioRxiv (for pre-print articles) was conducted to identify studies exploring genotype-phenotype correlation in subjects with XLHED. The search terms “*EDA* variant” or “genotype” and “hypohidrosis”, “sweating”, “thermoregulation” or “phenotype” were used in different combinations to identify the relevant records published till July 30, 2025. Our literature search was supplemented by screening the references listed in the relevant papers including both original research articles, case reports, and reviews. After identifying all the relevant studies, the publications were retrieved from the databases and downloaded locally, followed by data extraction on a worksheet. The following eligibility criteria were applied for inclusion of any report in the systematic review: article type (case-control study, cohort study, case report), population (human subjects with XLHED), genetic information (studies that provided genotype information on the patients), and language (publications in English only). Studies were rejected if they met one of the following exclusion criteria: duplicated findings, papers with insufficient data, or studies published more than 50 years ago. Two of the authors, AB and HS, independently reviewed the papers, focusing on sweat volumes obtained after standard pilocarpine iontophoresis and thermoregulatory ability of patients with XLHED. This systematic review was registered in the PROSPERO registry on September 16, 2025.

## 3 Results

### 3.1 Cohort study

The first standardised measurement of pilocarpine-induced sweating in our cohort of 14 individuals with hypomorphic *EDA* mutations yielded sweat volumes between 2.0 and 43.0 µL within 30 minutes (Table 1). Heat intolerance was reported by eight of these patients or their parents. In three more individuals with various hypomorphic mutations and sweat volumes up to 12 µL, clinical signs of thermoregulation problems were detected, although these were not perceived as heat intolerance (Table 1). Three other individuals with pathogenic *EDA* variants, who had produced more than 15 µL of sweat upon stimulation with pilocarpine, did not complain about heat intolerance and no signs of insufficient thermoregulation were evident (Table 1).

**Table 1:** Heat intolerance of subjects carrying hypomorphic *EDA* mutations.

Subject number	*EDA* variant	Age at first measurement of pilocarpine-induced sweating (years)	Sweat volume in this first test (µL)	Parental- or self-reported heat intolerance	Clinical signs of heat intolerance^a^
1	c.553_588del36 (p.N185_P196del)	35	2.0	yes	yes
2	c.536_571del36 (p.G180_P191del)	19	3.0	yes	yes
3	c.536_571del36 (p.G180_P191del)	3	4.0	yes	yes
4	c.826C > T (p.R276C)	12	5.0	yes	yes
5	c.533_552del-insCTGAA (p.K178_P184del-insTE)	12	5.0	yes	yes
6	c.784G > T (p.V262P)	33	5.5	yes	yes
7	c.533_552del-insCTGAA (p.K178_P184del-insTE)	7	6.0	yes	yes
8	c.1152G > C (p.R384S)	14	6.0	yes	yes
9	c.572_589del18 (p.P191_P196del)	29	10.0	no	yes
10	c.527G > T (splice site mutation)	6	11.0	no	yes
11	c.533_552del-insCTGAA (p.K178_P184del-insTE)	5	12.0	no	yes
12	c.935T > A (p.I312N)	2	16.0	**no**	**no**
13	c.948C > G (p.D316E)	32	29.0	**no**	**no**
14	c.769G > C (p.G257R)	25	43.0	**no**	**no**

a clinical signs, such as a head warm to the touch, irritability, feeling weak, syncopation, fever, or suffering from poor sleep in summer.

Bold text indicates the absence of any clinical signs.

In the youngest patient, subject #12, the first measurement of induced perspiration during infancy resulted in a sweat volume of 16 µL. Follow-up data until puberty confirmed sweat production at the lower end of the normal range. Pilocarpine-induced sweat volumes were 18 µL at the age of four years and 24 µL at the age of 10 years. The two adult patients #13 and #14 without clinical signs of heat intolerance showed even higher sweat volumes.

Normal body temperature regulation was observed both when 10-year-old subject #12 (26.9 kg body weight) had a sauna and during/after intense exercising (Table 2). He lost 0.33 kg body mass within 30 minutes in connection with the dry sauna, taking into account his interim fluid intake of 80 mL, and outperformance on a bicycle ergometer for 22 minutes led only to a slight and temporary elevation of body core temperature. Thus, a pilocarpine-induced sweat volume of 16 µL in early childhood correlated with normal thermoregulation at the age of 10 years.

**Table 2:** Thermal or exercise-induced sweating of subject #12 at the age of 10 years.

Stress condition	Time point	Maximum skin temperature^a^ (°C)	Body core temperature^b^ (°C)
*Controlled thermal stress in the sauna* (85°C for 12.5 min, weight loss of 0.33 kg within 30 minutes)	Before having a sauna	35.42	n.d.
	Directly after sauna	38.03	n.d.
	5 min after sauna	36.29	n.d.
	10 min after sauna	35.68	n.d.
	15 min after sauna	34.94	n.d.
*Continuous exercise on a bicycle ergometer for 22 minutes* (performance 3.0 W/kg; maximal heart rate 172/min)	Pre-exercise	n.d.	36.98
	30 min post exercise start	n.d.	37.37
	40 min post exercise start	n.d.	36.77
	60 min post exercise start	n.d.	36.53
	90 min post exercise start	n.d.	36.92

n.d., not determined.

a determined by infrared thermography.

b measured with an ingestible thermometer pill.

### 3.2 Literature review findings

As we were aware that other research groups had also investigated genotype-phenotype correlation in subjects with XLHED and may have used pilocarpine iontophoresis to assess the sweating ability, we carried out a systematic literature search in PubMed, Google Scholar, and Embase (for published articles), and bioRxiv (for pre-print articles) till July 30, 2025, which yielded 382 relevant records. Only 49 studies met all inclusion criteria and none of the exclusion criteria. Altogether, 23 studies were identified, which investigated genotype-phenotype correlations with regard to perspiration or thermoregulation in patients with known *EDA* variants. Among them, 6 studies provided data on standardised tests of pilocarpine-induced sweat production (7, 8, 16, 18–20), but only two of these papers, both from the Centre for Ectodermal Dysplasias in Erlangen, described a total of 7 patients with hypomorphic *EDA* mutations (7, 19) and pilocarpine-induced sweat volumes between 2.0 and 12.0 µL. All seven patients are included in Table 1. Their clinical signs of insufficient thermoregulation suggest that the recently reported sweat volumes in healthy infants (8, 9) with a minimum pilocarpine-induced sweat production of 15 µL/30 min in boys indeed represent the bottom of the normal range.

## 4 Discussion

Our cohort data indicate that adequate thermoregulatory capacity of male individuals is associated with a pilocarpine-induced sweat volume of 15 µL or above in the Macroduct sweat collector, when assessed during infancy. The systematic literature review supported this view, although it provided only very limited additional patient data. Nevertheless, it confirmed our empirical assumption that the pilocarpine-induced sweat volume does not increase significantly until the onset of puberty (7, 19), consistent with data from male patients who were treated prenatally with an ectodysplasin A1 replacement protein and followed up for 10 years (8; additional unpublished observations). A great difficulty of our study, however, is that it attempts to convert a continuous variable (sweat volume) into a binary endpoint (sufficient versus insufficient sweating). As sweat production varies by age, sex, ethnicity, body size, and environmental factors, the proposed threshold cannot be generalized. It refers to male infants who are ethnically Central or Western European and grow up in the Central European climate.

While there is no specific definition of heat intolerance, the concept has been well recognised and widely reported in the scientific literature. Using methods similar to those described here, heat intolerance has been subjectively reported or empirically demonstrated in individuals with XLHED (21, 22). However, minor problems with thermoregulation may not always be perceived as a relevant issue, although insufficient perspiration is supposed to have an impact on sleep quality and to make affected individuals feel tired during the day. It is also well known that men with XLHED retire earlier than unaffected individuals.

Humans have evolved to maintain their body core temperature within a narrow range of values. Resting temperatures of 36 to 37.5°C are typical. During exercise or ambient heat stress, transient elevations in body temperature to up to 40°C are normal and generally well tolerated. We have shown here, that when exposed to heat stress, subject #12 was able to thermoregulate normally and maintain his body core temperature within the normal range. This can be attributed to the subject's ability to sweat. In general, individuals with XLHED are unable to control their body temperature through dry routes of heat dissipation (conduction and convection) and only lose up to 6% of heat via evaporation, compared to 67% by the same route in unaffected individuals (22). Subject #12's body mass loss of 0.33 kg in response to thermal stress in a sauna also corresponds well to the mean values obtained from healthy young women (0.37 kg weight loss; 59.10 kg mean body weight) and healthy young men (0.50 kg weight loss; 75.98 kg mean body weight) under similar stress conditions (23).

In addition, the results reported here are in good agreement with those of a previous study on Central European patients with pathogenic *TP63* variants (24), which showed that pilocarpine-induced sweat volumes of 11 µL and below were associated with heat intolerance.

## 5 Conclusion

When taken together, our data and a systematic review of the available literature support the conclusion that a sweat volume ≥15 µL obtained by standardised sweat collection over 30 minutes after topical pilocarpine iontophoresis in infancy is a valid indicator of normal thermoregulatory ability of male individuals in Central or Western Europe.

## Data Availability

The original contributions presented in the study are publicly available. This data can be found here: (ClinVar submission ID: SUB16379238).

## References

[1] Blüschke G Nüsken KD Schneider H. Prevalence and prevention of severe complications of hypohidrotic ectodermal dysplasia in infancy. Early Hum Dev. (2010) 86:397–9. 10.1016/j.earlhumdev.2010.04.008 20682465

[2] Salisbury DM Stothers JK. Hypohidrotic ectodermal dysplasia and sudden infant death. Lancet. (1981) 1:153–4. 10.1016/s0140-6736(81)90736-4 6109826

[3] Prasun P Karmarkar SA Agarwal A Stockton DW. Unusual physical features and heat stroke presentation for hypohidrotic ectodermal dysplasia. Clin Dysmorphol. (2012) 21:24–6. 10.1097/MCD.0b013e32834cef61 21959862

[4] Hammersen J Neukam V Nüsken KD Schneider H. Systematic evaluation of exertional hyperthermia in children and adolescents with hypohidrotic ectodermal dysplasia: an observational study. Pediatr Res. (2011) 70:297–301. 10.1203/PDR.0b013e318227503b 21646941

[5] Kere J Srivastava AK Montonen O Zonana J Thomas N Ferguson B. X-linked anhidrotic (hypohidrotic) ectodermal dysplasia is caused by mutation in a novel transmembrane protein. Nat Genet. (1996) 13:409–16. 10.1038/ng0895-409 8696334

[6] Nguyen-Nielsen M Skovbo S Svaneby D Pedersen L Fryzek J. The prevalence of X-linked hypohidrotic ectodermal dysplasia (XLHED) in Denmark, 1995–2010. Eur J Med Genet. (2013) 56:236–42. 10.1016/j.ejmg.2013.01.012 23416623

[7] Schneider H Hammersen J Preisler-Adams S Huttner K Rascher W Bohring A. Sweating ability and genotype in individuals with X-linked hypohidrotic ectodermal dysplasia. J Med Genet. (2011) 48:426–32. 10.1136/jmg.2010.084012 21357618

[8] Schneider H Schweikl C Faschingbauer F Hadj-Rabia S Schneider P. A causal treatment for X-linked hypohidrotic ectodermal dysplasia: long-term results of short-term perinatal ectodysplasin A1 replacement. Int J Mol Sci. (2023) 24:7155. 10.3390/ijms24087155 37108325 PMC10138843

[9] Hadj-Rabia S Nguyen-Khoa T Mashiah J. Sweat volume quantification in paediatric population. J Eur Acad Dermatol Venereol. (2025) 39:e276–7. 10.1111/jdv.20255 39105533

[10] Tochihara Y Wakabayashi H Lee JY Wijayanto T Hashiguchi SM. How humans adapt to hot climates learned from the recent research on tropical indigenes. J Physiol Anthropol. (2022) 41:27. 10.1186/s40101-022-00302-3 35836266 PMC9281079

[11] Niu Z Goto T. Effects of individual characteristics and local body functions on sweating response: a review. Int J Biometeorol. (2024) 68:2185–204. 10.1007/s00484-024-02758-7 39141136 PMC11519300

[12] Lee JB Bae JS Matsumoto T Yang HM Min YK. Tropical Malaysians and temperate Koreans exhibit significant differences in sweating sensitivity in response to iontophoretically administered acetylcholine. Int J Biometeorol. (2009) 53:2149–57. 10.1007/s00484-008-0197-9 19048305

[13] Wünsche S Jüngert J Faschingbauer F Mommsen H Goecke T Schwanitz K. Noninvasive prenatal diagnosis of hypohidrotic ectodermal dysplasia by tooth germ sonography. Ultraschall Med. (2015) 36:381–5. 10.1055/s-0034-1384933 25140498

[14] Hammersen J Wohlfart S Goecke TW Köninger A Stepan H Gallinat R. Reliability of prenatal detection of X-linked hypohidrotic ectodermal dysplasia by tooth germ sonography. Prenat Diagn. (2019) 39:796–805. 10.1002/pd.5384 30394555

[15] Schneider H Schneider M Lia M Grange DK Hadj-Rabia S Clarke A. Attitudes of female carriers of X-linked hypohidrotic ectodermal dysplasia towards prenatal treatment and their decisions during a pregnancy with a male fetus. Orphanet J Rare Dis. (2025) 20:182. 10.1186/s13023-025-03710-7 40234959 PMC12001694

[16] Schneider H Faschingbauer F Schuepbach-Mallepell S Körber I Wohlfart S Dick A. Prenatal correction of X-linked hypohidrotic ectodermal dysplasia. N Engl J Med. (2018) 378:1604–10. 10.1056/NEJMoa1714322 29694819

[17] Schneider H Hadj-Rabia S Faschingbauer F Bodemer C Grange DK Norton ME. Protocol for the phase 2 EDELIFE trial investigating the efficacy and safety of intra-amniotic ER004 administration to male subjects with X-linked hypohidrotic ectodermal dysplasia. Genes (Basel). (2023) 14:153. 10.3390/genes14010153 36672894 PMC9858920

[18] Jones KB Goodwin AF Landan M Seidel K Tran D-K Hogue J. Characterization of X-linked hypohidrotic ectodermal dysplasia (XL-HED) hair and sweat gland phenotypes using phototrichogram analysis and live confocal imaging. Am J Med Genet Part A. (2013) 161:1585–93. 10.1002/ajmg.a.35959 PMC4414120 23687000

[19] Burger K Schneider A-T Wohlfart S Kiesewetter F Huttner K Johnson R. Genotype-phenotype correlation in boys with X-linked hypohidrotic ectodermal dysplasia. Am J Med Genet Part A. (2014) 164:2424–32. 10.1002/ajmg.a.36541 24715423

[20] Gökdere S Schneider H Hehr U Willen L Schneider P Maier-Wohlfart S. Functional and clinical analysis of five EDA variants associated with ectodermal dysplasia but with a hard-to-predict significance. Front Genet. (2022) 13:934395. 10.3389/fgene.2022.934395 35923710 PMC9339965

[21] Rietschel RL Wilmore DW. Heat loss in anhidrotic ectodermal dysplasia. J Invest Dermatol. (1978) 71:145–7. 10.1111/1523-1747.ep12546880 681783

[22] Massey H House J Tipton M. Thermoregulation in ectodermal dysplasia: a case series. Int J Environ Res Public Health. (2019) 16:4514. 10.3390/ijerph16224514 31731639 PMC6888138

[23] Podstawski R Boraczyński T Boraczyński M Choszcz D Mańkowski S Markowski P. Sauna-induced body mass loss in young sedentary women and men. Sci World J. (2014) 2014:307421. 10.1155/2014/307421 PMC4295591 25614882

[24] Ferstl P Wohlfart S Schneider H. Sweating ability of patients with p63-associated syndromes. Eur J Pediatr. (2018) 177:1727–31. 10.1007/s00431-018-3227-6 30088137

